# A Comparative Study on Evolutionary Multi-objective Optimization Algorithms Estimating Surface Duct

**DOI:** 10.3390/s18124428

**Published:** 2018-12-14

**Authors:** Qixiang Liao, Zheng Sheng, Hanqing Shi, Lei Zhang, Lesong Zhou, Wei Ge, Zhiyong Long

**Affiliations:** College of Meteorology and Oceanology, National University of Defense Technology, Nanjing 211101, China; liaoqixiang2013@e-mail.com (Q.L.); zlei_best@126.com (L.Z.); 17327729696@163.com (L.Z.); gewei_NUDT@163.com (W.G.); longzy@aliyun.com (Z.L.)

**Keywords:** multi-objective optimization algorithm, atmospheric duct, GPS, hypervolume, inverted generational distance

## Abstract

The problem of atmospheric duct inversion is usually solved as a single objective optimization problem. Based on ground-based Global Positioning System (GPS) phase delay and propagation loss, this paper develops a multi-objective method including the effect of source frequency and receiving antenna height. The diversity and convergence of solution sets are evaluated for seven multi-objective evolutionary algorithms with three performance metrics: Hypervolume (HV), Inverted Generational Distance (IGD), and the averaged Hausdorff distance ($\Delta_{2}$). The inversion results are compared with the simulation results, and the experimental comparison is conducted on three groups of test situations. The results demonstrate that the ranking of algorithm performance varies because of the different methods used to calculate performance metrics. Moreover, when the algorithms show overwhelming performance using performance metrics, the inversion result is not more close to the real value. In the comparison of computational experiments, it was found that, as the retrieved parameter dimension increases, the inversion result becomes more unstable. When the observed data are sufficient, the inversion result seems to be improved.

## 1. Introduction

Atmospheric duct is a layer of the atmosphere that causes electromagnetic waves to bend toward the earth’s surface. The inversion of atmospheric duct structure affects the normal operation of electromagnetic weapon and communication systems. Therefore, various methods of sounding refractivity profiles have been put forward [1,2,3,4,5]. Based on the relations between radar echo and refractivity, the technique of refractivity from clutter (RFC) was developed. The technique changes the method of detecting atmospheric duct. Subsequent studies by Gerstoft [6,7], Yardim [8], Sheng [9], and other scholars [10,11,12] have been mainly concerned with improving the inversion algorithm. When Hitney [13] demonstrated the capability of estimating the trapping layers base height from ultrahigh frequency (UHF) signal strengths, the method of remote sensing refractivity profiles from Global Positioning System (GPS) signals was developed. Anderson [14] tried to infer atmospheric duct with phase delay, but there was a certain gap between the inversion results and the real refractivity profile. Liao et al. presented the inversion method of low-elevation ground-based GPS, combining phase delay and propagation loss information to retrieve surface duct parameters, and recently proposed the non-dominated sorting genetic algorithm II combined with simulated annealing algorithm (NSSAGA) with matching fields to improve inversion results [15,16]. However, they neglected to consider the influence of source frequency. When they designed the cost function, all antenna information was combined into two objective functions. Hence the advantages of a multi-objective algorithm were not fully exploited.

In multi-objective optimization problems, the objective function usually has the characteristics of being multi-peak, discontinuous, non-differential and even discrete. Scholars have tried to adopt different random search and heuristic algorithms, such as genetic algorithm (GA), differential evolution, and particle swarm optimization [17,18,19,20]. In the development of these algorithms, evolutionary computation has been proved to have good performance. After the GA was first applied to multi-objective optimization problems, various multiobjective evolutionary algorithms (MOEA) are developed. The classic MOEA includes NSGA, NSGA-II, improved MOEA (MOEAD) and other algorithms [21,22,23,24,25]. These algorithms can be divided into five categories according to their characteristics: decomposition-based approaches; indicator-based approaches; approaches that modify the secondary diversity-based selection criteria; approaches that modify the traditional dominance relation; and preference-based approaches. Li et al. tested various state-of-the-art multi-objective algorithms, and the results showed that the algorithm performance was not consistent among different problems [25]. Therefore, an appropriate multi-objective algorithm should be selected according to the specific problem.

Based on these conclusions, this paper proposes a method of testing the performance of five kinds of multi-objective algorithms on the jointed inversion problem of atmospheric duct when using GPS data, in order to find the optimal algorithm that is most suitable for such problems. This paper uses Hypervolume (HV), Inverted Generational Distance (IGD) and the averaged Hausdorff distance, metrics to evaluate the characteristics of optimization algorithms in the test process. The three metrics can reflect the convergence and diversity of solution sets. Additionally, this paper compares the simulation profile and inversion results of seven algorithms under different test conditions and evaluates the accuracy of the inversion results.

The rest of this paper is organized as follows. Section 2 introduces key concepts in the field of evolutionary algorithms and the methods of calculating phase delay and propagation loss. How to parameterize surface duct is also explained in this section. The comparison of seven multi-objective algorithms on the atmospheric duct inversion problem is the subject of Section 3, which itself is divided into three parts: excluding the effect of source frequency and receiving antenna height; considering the effect of source frequency; and considering the effect of both. The last section offers concluding remarks and future perspectives.

## 2. Multi-Objective Optimization Using Evolutionary Algorithms

### 2.1. Basic Concepts of Multi-Objective Optimization

In multi-objective optimization problems, there are usually *M* conflicting optimization goals. To express the concept of optimization, such problems can be expressed as [25]:

(1) $$F\left(\vec{X}\right)=\min\left\{f_{1}\left(\vec{X}\right),f_{2}\left(\vec{X}\right),f_{3}\left(\vec{X}\right)\cdots ,f_{M}\left(\vec{X}\right)\right\}$$

In (1), $\vec{X}$ represents the decision vectors ${\left(x_{1},x_{2},x_{3},\cdots ,x_{D}\right)}^{T}$, where *D* is the number of decision variables. Meanwhile, the decision vectors $\vec{X}$ should satisfy the constraint conditions:

(2) $$\Omega =\left\{\vec{X}|\vec{X}\in R^{D},g_{i}\left(\vec{X}\right)\leq 0,h_{j}\left(\vec{X}\right)\equiv 0,i=1,2,\ldots ,k;j=1,2,\ldots ,l\right\}$$

Here, $g_{i}\left(\vec{X}\right)\leq 0\left(i=1,2,\ldots ,k\right)$ is the inequality constraint, and $h_{j}\left(\vec{X}\right)\equiv 0\left(j=1,2,\ldots ,l\right)$ is the equality constraint. In most cases, all components of objective functions vector cannot be minimized simultaneously. Accordingly, the Pareto front needs to be introduced to obtain the optimal decision variables.

Suppose there are two decision variables, ${\vec{X}}_{a}$ and ${\vec{X}}_{b}$, then if, and only if, ∀i∈{1,2,…,M} there are $f_{i}\left({\vec{X}}_{a}\right)\leq f_{i}\left({\vec{X}}_{b}\right)$, and ∃j∈{1,2,…,M} that makes $f_{j}\left({\vec{X}}_{a}\right)≺f_{j}\left({\vec{X}}_{b}\right)$, signed ${\vec{X}}_{a}≺{\vec{X}}_{b}$. The non-dominated set of the entire feasible decision is called the Pareto optimal solution set (PS), and the boundary defined by the set of all point mapped from the Pareto optimal set is called the Pareto front (PF).

### 2.2. Evaluation Metrics

Evolutionary algorithms perform well in finding multi-objective solutions and can ultimately obtain a non-dominated solution set. Therefore, how to evaluate the performance of these algorithms is also extremely important. There are two ways to judge the quality of non-dominated solution sets. First, after the decision space is projected into the target space, it is dependent on the distance between the retrieved PF and the actual PF surface. The smaller the distance, the better the convergence. Second, it depends on the distribution of the PF surface. The more uniform the distribution, the better the PF. In this paper, three metrics are used to evaluate the performance of five kinds of multi-objective algorithms on surface duct inversion.

1. Hypervolume

Zitzler and Thiele proposed HV in 1999, and it has been widely used since 2003 [26,27]. HV is used to calculate the hypervolume of a non-dominated set and space reference point, which can be simply constructed as the solution set corresponding to the worst target vector. The expression is as follows:

(3) $$\mathrm{HV}=volume\left(\overset{\underset{\left|Q\right|}{i=1}}{U}v_{i}\right)$$

Here, Q is the solution set of the non-dominant solution front, and |Q| is the number of non-dominant solution elements. For each non-dominated solution i∈Q, a hypercube $v_{i}$ is composed of reference points and members i. Larger metric values indicate that the Pareto solution set obtained can be more widely distributed. Therefore, the bigger the value, the better the algorithm performance.

2. Inverted Generational Distance

IGD is the reversal of Generational Distance (GD). GD was originally proposed to evaluate the distance between a non-dominated solution set and the real PS. It is an index to evaluate the convergence. IGD is the average value of the minimum distance between the uniform point on the PF and the non-dominated solution set. The calculation is as follows [28]:

(4) $$\mathrm{IGD}\left(P^{*},\mathrm{NDS}\right)=\frac{\sum_{i=1}^{\left|P^{*}\right|}d\left(i,\mathrm{NDS}\right)}{\left|P^{*}\right|}$$

(5) $$d\left(i,NDS\right)=\overset{\left|A\right|}{\underset{j=1}{\min}}\sqrt{\sum_{m=1}^{M}{\left(\frac{f_{m}(p_{i})-f_{{}_{m}}(a_{j})}{f_{m}^{\max}-f_{m}^{\min}}\right)}^{2}}$$

Here, $P^{*}=\left\{p_{1},p_{2},\ldots ,p_{w}\right\}$ represents the uniform points covering the optimal PF surface, and d(i,NDS) is the minimum Euclidean distance from the point *i* to the individual in the non-dominated solution set (NDS). *A* is the approximate Pareto solution set obtained by the algorithm. $f_{m}^{max}$ and $f_{m}^{min}$ are the maximum and minimum respectively of the *m*-th objective function values of the real solution set $P^{*}$. $\left|p^{*}\right|$ and |A| are the number of the real solution set elements and the approximate Pareto solution set elements, respectively. Since $P^{*}$ can represent the real PF of the whole problem, the convergence and diversity of the PF obtained is better when the IGD value is lower.

**Figure 1:**
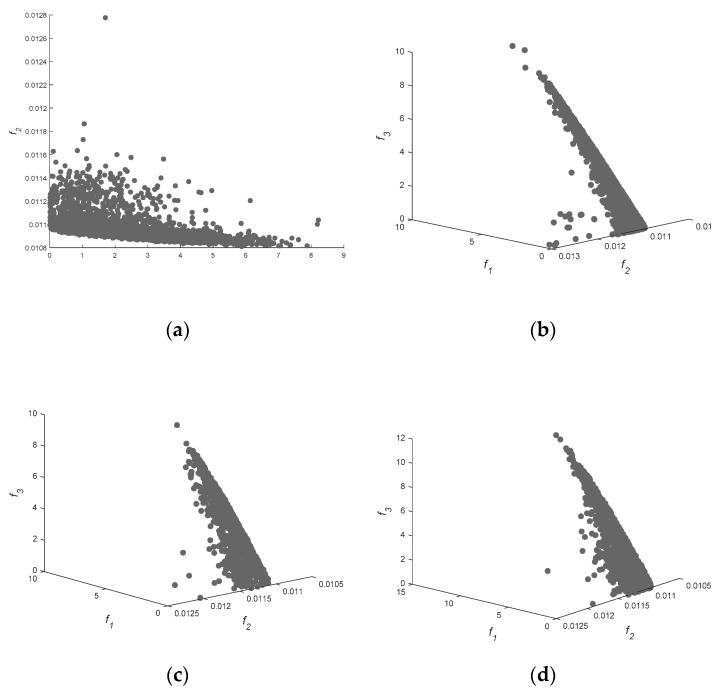
The distribution of reference point set for different solution spaces. Subfigure (**a**–**d**) are different real PFs for different problems, such as GPS1, GPS2, GPS3, GPS4 and GPS5.

Considering that the IGD value is related to the real PF, we set different real PFs for different problems (seeing Figure 1). The solution space can be divided into two, four, six and eight dimensions according to the height of receiving antenna and the frequency of transmitting source during surface duct inversion, marking the different problems as GPS1, GPS2, GPS3, GPS4 and GPS5. When the solution space is six dimensions, it can be divided into two types of problem, GPS3 and GPS4. The solution of the GPS3 condition is to adopt two sets of data from the same height and different frequencies, and a set of data from other heights. The solution of the GPS4 condition is to consider three sets of data from different heights and frequencies. The contribution of those real PFs to the HV is very small, and IGD is less sensitive to the PF simplification [29]. Therefore, a reference point set is constructed as the real PF by selecting non-dominated solutions from all the obtained solutions by 30 runs ofeach algorithm.

3. The Averaged Hausdorff Distance

An ‘averaged Hausdorff distance’, $\Delta_{s}$, was first proposed as one possible remedy for the Hausdorff distance [30,31]. The indicator averages the distances between entries of outcome set and Pareto front approximation. Hence, it is more suitable for multiobjective evolutionary algorithms because of single (or few) outliers generated. $\Delta_{s}$ is composed of the modified indicators GD and IGD. Let $Q=\left\{y_{1},\ldots ,y_{n}\right\}$, $p^{*}=\left\{p_{1},\ldots ,p_{w}\right\}\subset$R^k^ be non-empty and finite, then

(6) $$\Delta_{s}\left(Q,p^{*}\right)=max(\left(\frac{1}{N}\overset{N}{\underset{i=1}{\sum }}dist{\left(y_{i},Q\right)}^{s}{)}^{1/s},\frac{1}{M}\overset{M}{\underset{i=1}{\sum }}dist{\left(p_{i},Q\right)}^{s}{)}^{1/s}\right)$$

Due to its composition of modified *GD* and *IGD*, $\Delta_{s}$ has stronger metric properties than *IGD* [30]. Refering to [30], it is concluded that the smaller *s*, the easier single outliers will be punished by $\Delta_{s}$. Here, we focuse on the $\Delta_{2}$ scenario, i.e., the Euclidean norm. 

### 2.3. Phase-Delay Model and Propagation-Loss

There are two main effects when GPS signals pass through the troposphere. First, the travel speed of the signals becomes slower in the atmosphere. Second, the path of GPS signals bends. Both delays are caused by changes in the atmospheric refractivity, which can be expressed by Smith and Weintraub’s equation [32]:

(7) $$N=77.6\times p/T+3.73\times 10^{5}\times \left(e/T^{2}\right)$$

Here, the unit of T is *K*, and the unit of P and e is hPa. Assuming a spherically symmetric atmosphere, Sheng et al. presented the concrete calculation process of phase delay [33]. The excess phase path is defined as:

(8) $$\Delta S=S-S_{0}$$

The phase path S is determined as:

(9) $$S=\int_{r_{1}}^{r_{2}}r\cdot n^{2}/\sqrt{r^{2}\cdot n^{2}-a^{2}}dr=\int_{x_{1}}^{x_{2}}x\cdot \left[1-\left(x/n\right)dn/dx\right]/\sqrt{x^{2}-a^{2}}dx$$

In (9), x=rn(r) denotes the refractive radius. The ray path in a vacuum $S_{0}$ can be determined as:

(10) $$S_{0}=\sqrt{r_{1}^{2}+r_{2}^{2}-2r_{1}\cdot r_{2}\cdot cos\theta }$$

To model the GPS signal propagation, the single-way propagation loss L(x,z) of ground-based GPS signals is used to describe the objective function [33]:

(11) L(x,z)=20log(f)+20log(x)+C

In (11), f represents the propagation factor and C is a constant parameter. In a rectangular coordinate system, the propagation can be calculated as:

(12) $$f=\sqrt{x}\left|u\left(x,z\right)\right|$$

If the initial field is provided, the split-step Fourier transform (SSFT) is used to calculate low-elevation GPS signal propagation in a tropospheric duct. This can be substituted by a sine or cosine transform because the bottom boundary approximates to a perfectly conducting surface. The SSFT solution of the propagated wave equation is expressed as [16]:

(13) $$u\left(x+\Delta x,z\right)=exp(ik\left(M^{2}-1\right)\Delta x/2)\cdot F^{-1}\{exp(-i\pi^{2}p^{2}\Delta x/\left(2k\right))F\left[u\left(x,z\right)\right]\}$$

In (13), F and $F^{-1}$ are the Fourier transform and inverse Fourier transform, respectively. Here, Δx is the range step, p=2ksinθ represents the transform variable for which θ is the angle from the horizontal, and u(x,z) is the initial field. Balvedi and Walter [34] describe a more detailed process for solving the parabolic equation.

The modified refractivity M, which considers the Earth’s curvature, is related to the radio refractivity N as follows [16]:

(14) M=N+0.157×r

Differentiating with respect to r, we obtain:

(15) dM/dr=dN/dr+0.157

In the above, r is the altitude in meters and M is the modified refractivity. The unit of dM/dr is M-units/km. When the modified refractivity gradient is less than 0 M-units/km, an atmospheric duct occurs. The atmospheric structure, especially a surface duct, may delay the GPS signal at low elevation. Hence, this paper uses the GPS phase delay and propagation loss to retrieve the surface duct.

### 2.4. Parameterized Model

The atmospheric refractivity profile can be simulated by a parameterized model. The influence of a surface duct on electromagnetic wave propagation is more significant than that of an evaporation duct, and is usually represented by a three-segment linear model [16]:

(16) $$M\left(z\right)=M_{0}+\{\begin{array}{c} c_{1}z\\ c_{1}h_{1}+c_{2}\left(z-h_{1}\right)\\ c_{1}h_{1}+c_{2}h_{2}+0.118z \end{array}\begin{array}{c} z<h_{1}\\ h_{1}<z<h_{1}+h_{2}\\ z>h_{1}+h_{2} \end{array}$$

Here, $M_{0}$ is the corrected refractivity at sea level, $h_{1}$ is the trapping-layer base height, $c_{1}$ is the base slope, $h_{2}$ is the inversion-layer thickness, and $c_{2}$ is the slope from $h_{1}$ to $h_{1}+h_{2}$. The slope of the top layer is 0.118 M-units/m if we assume that the layer satisfies the standard refractivity condition.

## 3. Performance Comparison

To reconstruct the refractivity profile, a multi-objective cost function related to the ordinary least-squares (OLS) is defined [15,16]. The simulations assume a GPS elevation angle of 1° and a beam width of 16°. Here, we select seven algorithms for a computational test according to the characteristics of evolutionary algorithms. The specific parameter settings for each algorithm are shown in Table 1.

Table 2 shows the parameter settings for all test problems. In Table 2, GPS1, GPS2, GPS3 and GPS5 represent the problems when the solution space is two, four, six and eight dimensions, respectively, and the upper and lower bounds of the decision space are also given. The decision space includes slope parameters *c*_1_ and *c*_2_, duct height *h*_1_ and duct thickness *h*_2_, the transmitting frequency and receiving antenna height. In the sixth column, [1200, 1600] is the range of transmitting frequency, and [0, 200] is the range of antenna height. They are needed in Section 3.2 and Section 3.3 above. The first number is transmitting frequency, and the second number is antenna height in parentheses. In the computational test, although the solution space of GPS4 is also six dimensions, the received signal is from a different frequency at the same antenna height. The received signals of GPS3 are from two sets of antennas at the same height, which receive signals from different transmitting frequencies.

In the following, we compare the performance of seven algorithms after 30 runs of each algorithm, and present a box diagram and table of the evaluation metrics. We also compare the inversion value with the real value.

### 3.1. Excluding Antenna Height and Transmitting Frequency

**Figure 2:**
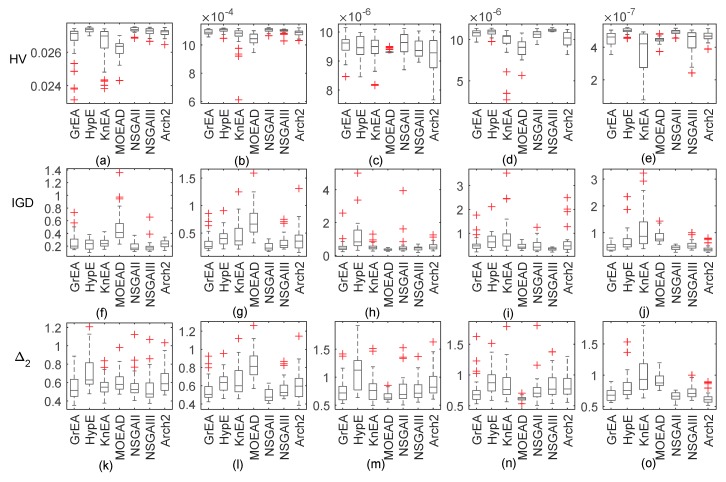
Box plots of the distribution of HV, IGD, and $\Delta_{2}$ values for the inversion problems with 7 test algorithms. Subfigure (**a**) to (**e**) are the boxplots of HV. Subfigure (**f**) to (**j**) are the boxplots of IGD. Subfigure (**k**) to (**o**) are the boxplots of $\Delta_{2}$.

Firstly, if we know the antenna height and transmitting frequency, then we only need to retrieve the parameter of refractivity profile. Figure 2 shows box plots representing the distribution of three metrics after 30 runs of each algorithm. A box plot (or box and whisker diagram) is a standardized way of displaying the distribution of data based on the five number summary: minimum, first quartile, median, third quartile, and maximum. In the simplest box plot the central rectangle spans the first quartile to the third quartile (the interquartile range or IQR). A segment inside the rectangle shows the median, and ’whiskers’ above and below the box show the locations of the minimum and maximum. Outlying values are marked as ‘+’.

As Figure 2 shows, the conventional NSGA-II and NSGA-III both perform well on surface duct inversion with HV and IGD as metrics. MOEAD performs poorly on problem GPS1 and problem GPS2. In other problems, if HV is used as the evaluation index, the algorithm performs poorly. However, if IGD is used as the evaluation index, the algorithm performs well. With HV as an indicator, HypE and GrEA perform similarly and both exhibit good performance. With IGD as an indicator, HypE is significantly inferior to GrEA. The reason for the difference in HV and IGD can be found in [25]. If the convergence of the PF has no difficulty, then the performance of the algorithm depends mainly on the solution distribution. However, HV and IGD use different methods to calculate solution distribution diversity. Therefore, if HV and IGD are taken as indicators, there will inevitably be inconsistent results. Meanwhile, it can be noticed that $\Delta_{2}$ almost has similary variation characteristics. We will only give the statistical results of $\Delta_{2}$ instead of IGD in the followed tables.

Table 3 and Table 4 show the mean and variance respectively of the HV and $\Delta_{2}$ metrics. In these tables, *M* represents the number of cost functions (solution space), and *D* represents the number of parameters to be retrieved (decision space). Excluding antenna height and transmitting frequency as inversion parameters is marked as condition 4, and including transmitting frequency is marked as 5. Including both is marked as 6. The gray background in the table indicates that the algorithm performs well in the inversion process, and the boldface indicates that the performance is poor.

It can be seen from Table 3 and Table 4 that when the decision space is under the same dimension, HV gradually decreases while $\Delta_{2}$ gradually increases as the dimension of the solution space increases. It shows that the poor definition of the inverse problem is enhanced, and that the evolutionary algorithm has difficulties converging to the real solution and obtaining more possible solutions. When we use HV as an indicator, HypE and NSGA-II are significantly superior to other algorithms in GPS inversion problems. MOEAD, by contrast, has an absolute disadvantage, which is roughly in line with the box plots. Some algorithms also perform well in specific problems, such as NSGA-III. Although there is no gray area in 15 problems, HV values are similar to NSGA-II in most cases. However, if $\Delta_{2}$ is used as the performance index, the conclusion is different. The performance of NSGA-II are consistent with Table 3, and there are more bad behavior with HypE’s performance. This may be due to differences in HV and $\Delta_{2}$ diversity assessment mechanisms, as can be inferred from their calculation formulae.

**Figure 3:**
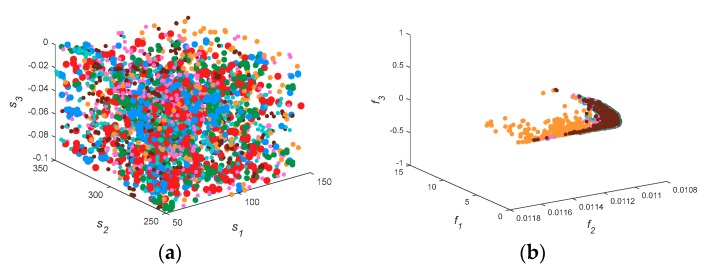

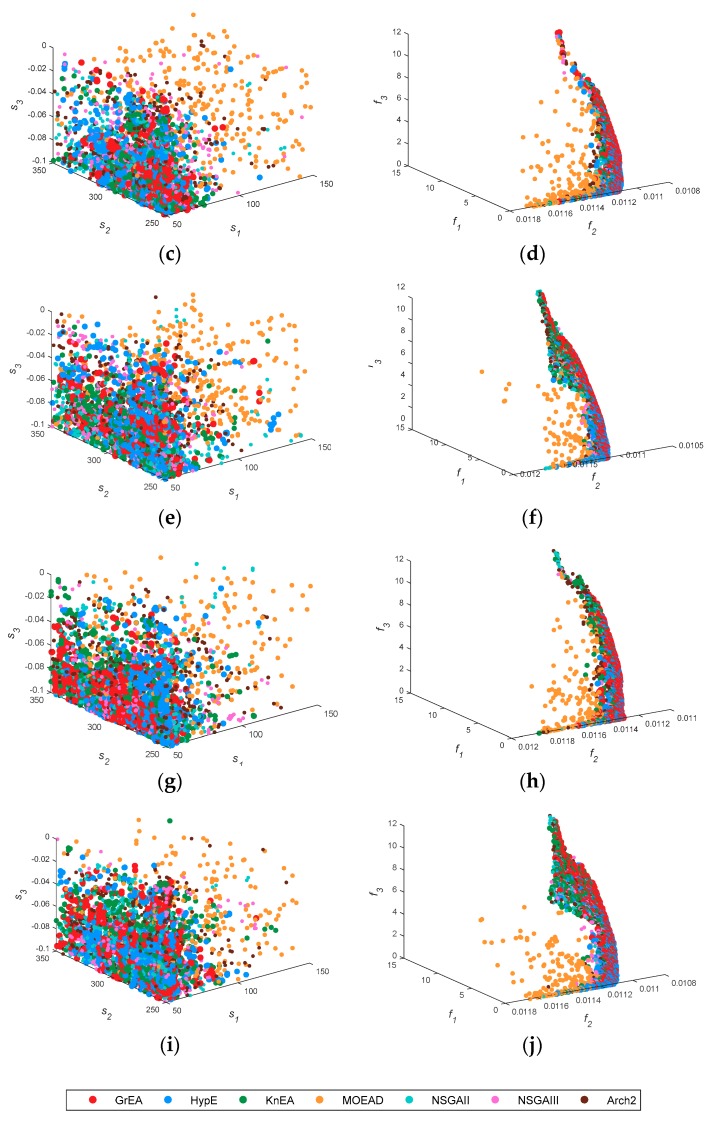
The spatial distribution of decision space and solution space in 7 test algorithms. Subfigure (**a**) to (**j**) are the distribution graph of decision space and solution space in GPS1, GPS2, GPS3, GPS4, and GPS5, respectively.

Figure 3 shows the spatial distribution of decision space and solution space, where the left plane is the three-dimensional distribution of the first three parameters, and the right plane is the distribution of the first three cost functions. Figure 3b shows the solution distribution of two-objective functions. It can be seen that the distribution of MOEAD is scattered, and that other algorithms approach the real value.

Therefore, it is more reasonable to evaluate with IGD. In Figure 3a, the solution sets of the seven algorithms are very scattered, indicating that the diversity and convergence of all multi-objective functions are more similar in the two-dimensional problem. The results are consistent with those in Table 3 and Table 4. KnEA, marked as green points, diffuses to the top and back of the PF in Figure 3f,h,j, and is confined in a narrow space. This phenomenon indicates that the performance of KnEA is poor, and that the solution space diversity is monotonous. If we compare the solution space alone, MOEAD and HypE may outperform other algorithms in surface duct inversion problems.

**Figure 4:**
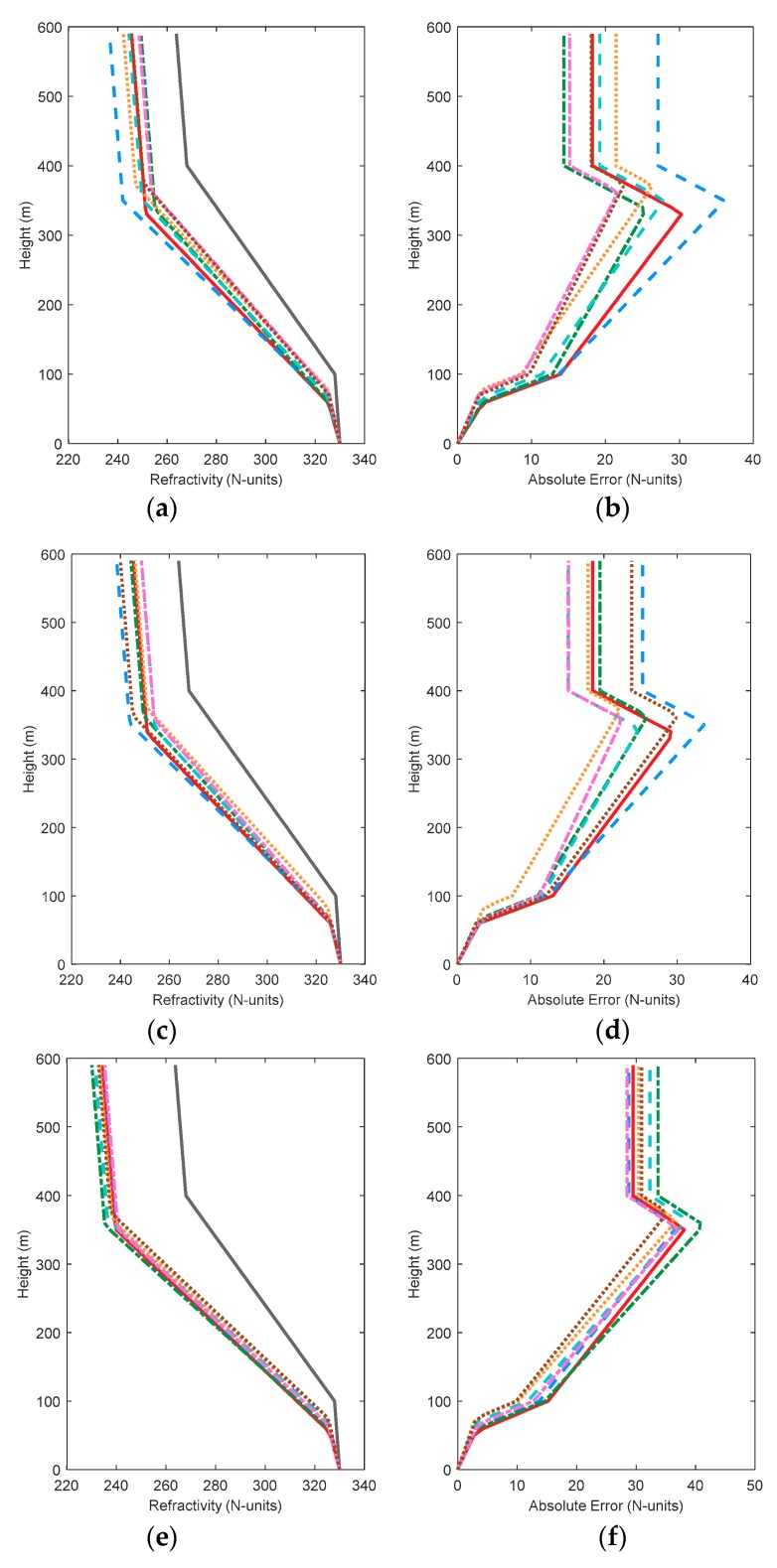

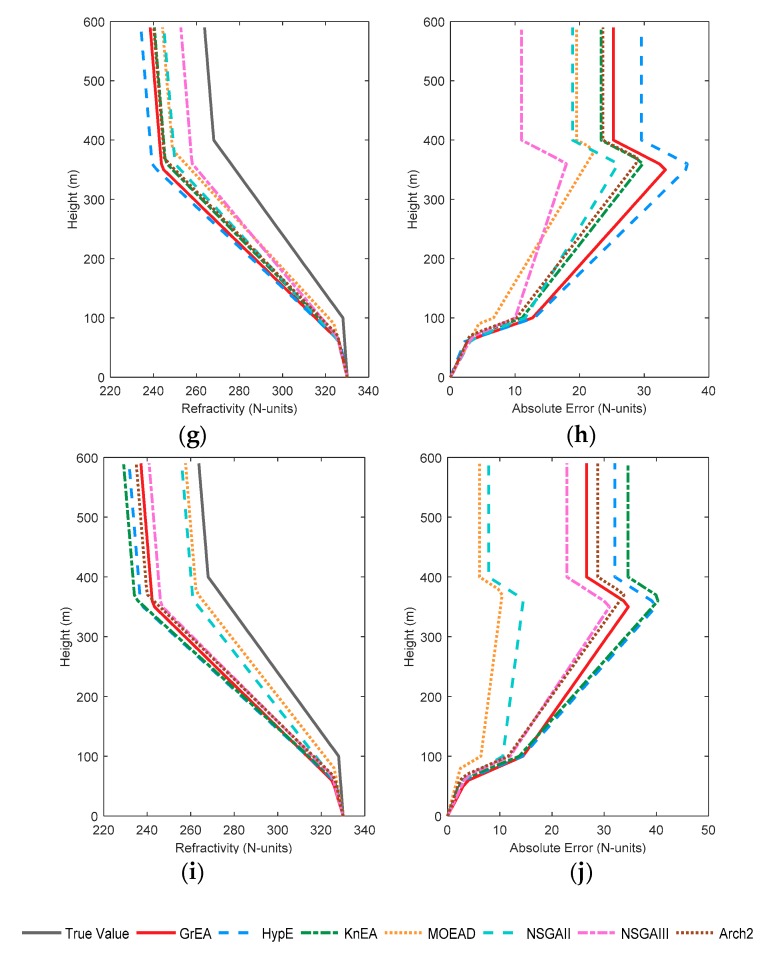
The comparison of simulated and retrieved profiles with 7 test algorithms. Subfigure (**a**) to (**j**) are the comparison diagram of simulated and retrieved profiles in GPS1, GPS2, GPS3, GPS4, and GPS5, respectively.

Figure 4 shows the comparison of simulated and retrieved profiles. The absolute errors between the inversion and simulated atmospheric refractivity profiles are given on the right plane. Although HypE performs well in performance tests, the results still show a gap between retrieved and simulated value, and are not as accurate as other algorithms. This indicates that HypE still has room for improvement. By contrast, MOEAD and NSGA-III can construct atmospheric refractivity profiles with relative accuracy in various conditions, indicating that they are more robust. Although KnEA obtained the worst metrics values and the worst retrieved results in the two-dimensional case, the inversion results occupy the middle level in other cases. The comparison of inversion results shows that the evaluation metrics can only evaluate the distribution of algorithms in decision space and solution space, but cannot be used to judge the actual inversion result directly. Therefore, it is necessary to select an appropriate algorithm according to the actual requirements in practical problems. By comparing Figure 4b,d,h,j, we can see whether an individual algorithm is improving step by step with an increase in the amount of observed data, such as MOEAD. However, in the GPS4 problem, the inversion results of MOEAD are no better than those of previous results.

### 3.2. Including Transmitting Frequency

**Figure 5:**
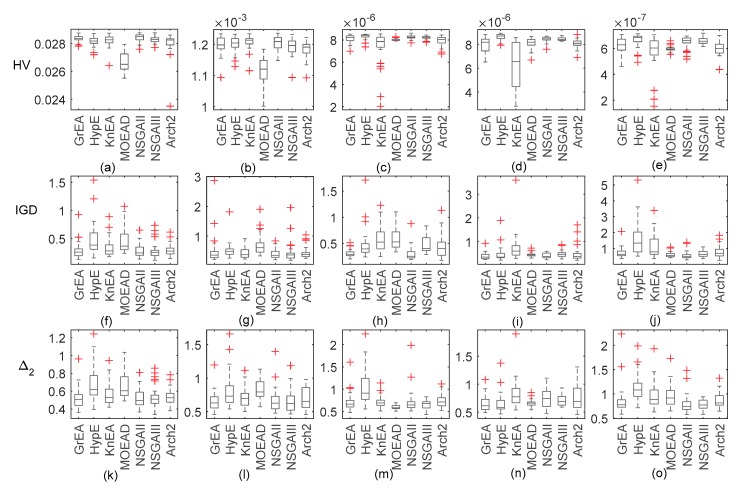
Box plots of the distribution of HV, IGD, and $\Delta_{2}$ values for the inversion problems with 7 test algorithms including transmitting frequency as an inversion parameter.Subfigure (**a**) to (**e**) are the boxplots of HV. Subfigure (**f**) to (**j**) are the boxplots of IGD. Subfigure (**k**) to (**o**) are the boxplots of $\Delta_{2}$.

Given that microwave signals of unknown frequencies are sometimes received, we can try to retrieve the atmospheric duct by collecting these signals. Therefore, transmitting frequency is also used as an inversion parameter in this subsection. Figure 5 shows box plots with five dimensions of decision space. NSGA-II remains the top performer with the HV metrics, followed by NSGA-III. MOEAD is the worst performer, followed by KnEA and GrEA. With IGD or $\Delta_{2}$ as the evaluation metric, similar conclusions can be obtained. However, MOEAD does not obtain the worst $\Delta_{2}$ values in problem GPS4 and problem GPS5. KnEA achieves the worst $\Delta_{2}$ values in problem GPS4, and HypE obtains the worst $\Delta_{2}$ values in problem GPS5.

**Figure 6:**
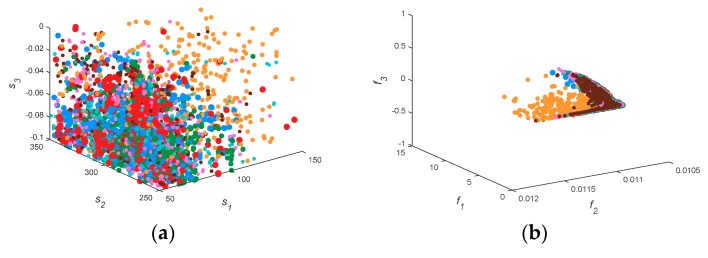

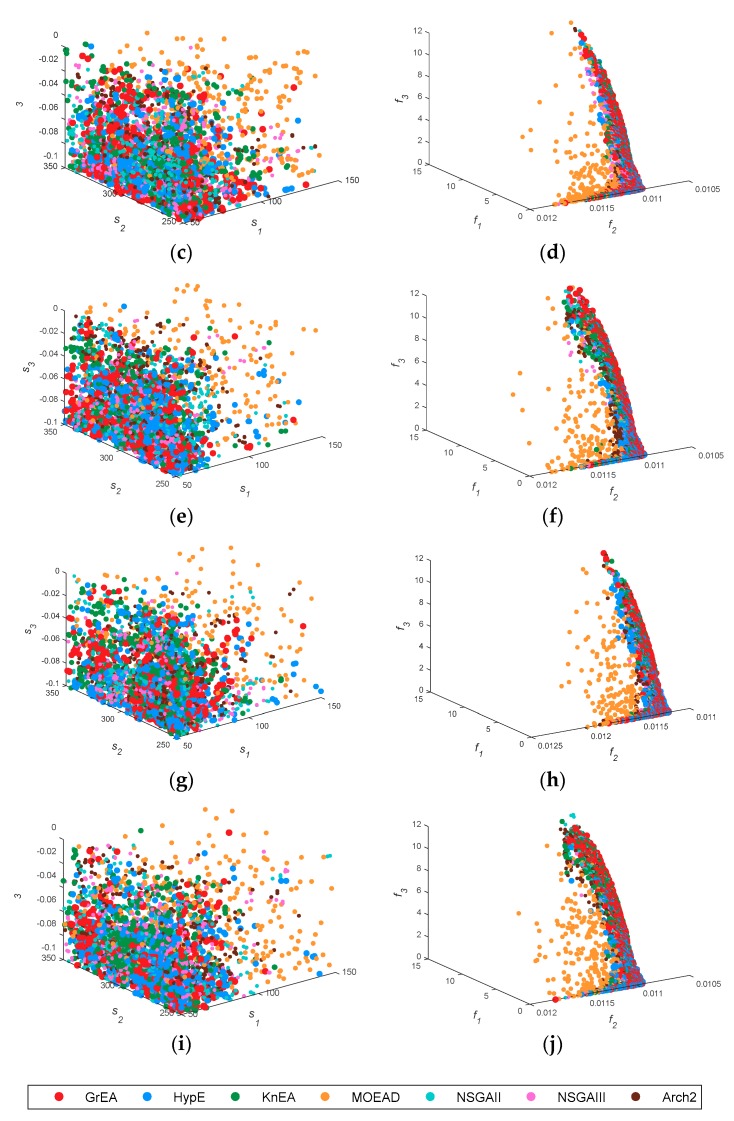
The spatial distribution of decision space and solution space in 7 test algorithms including transmitting frequency as an inversion parameter. Subfigure (**a**) to (**j**) are the distribution graph of decision space and solution space in GPS1, GPS2, GPS3, GPS4, and GPS5, respectively.

**Figure 7:**
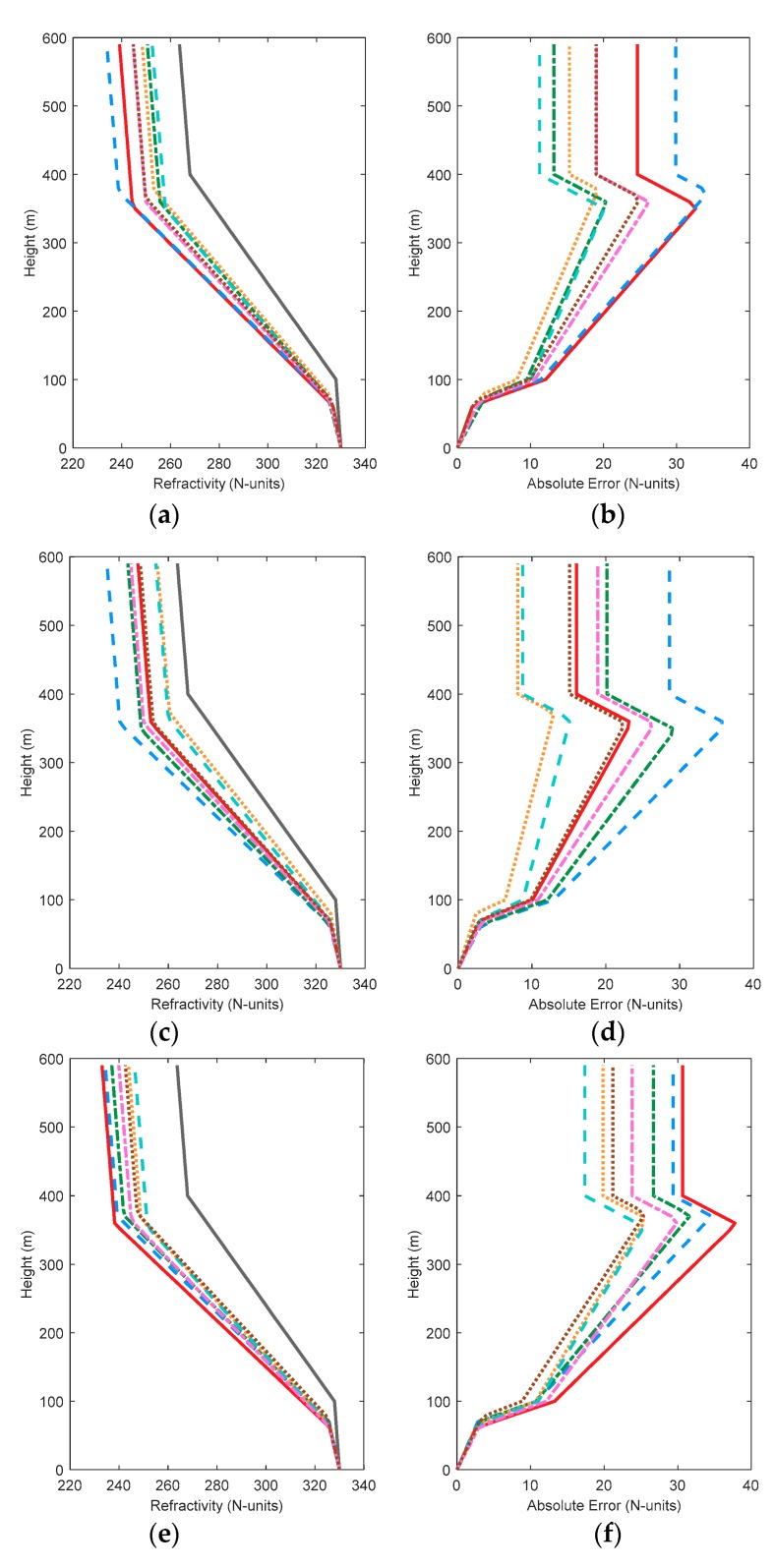

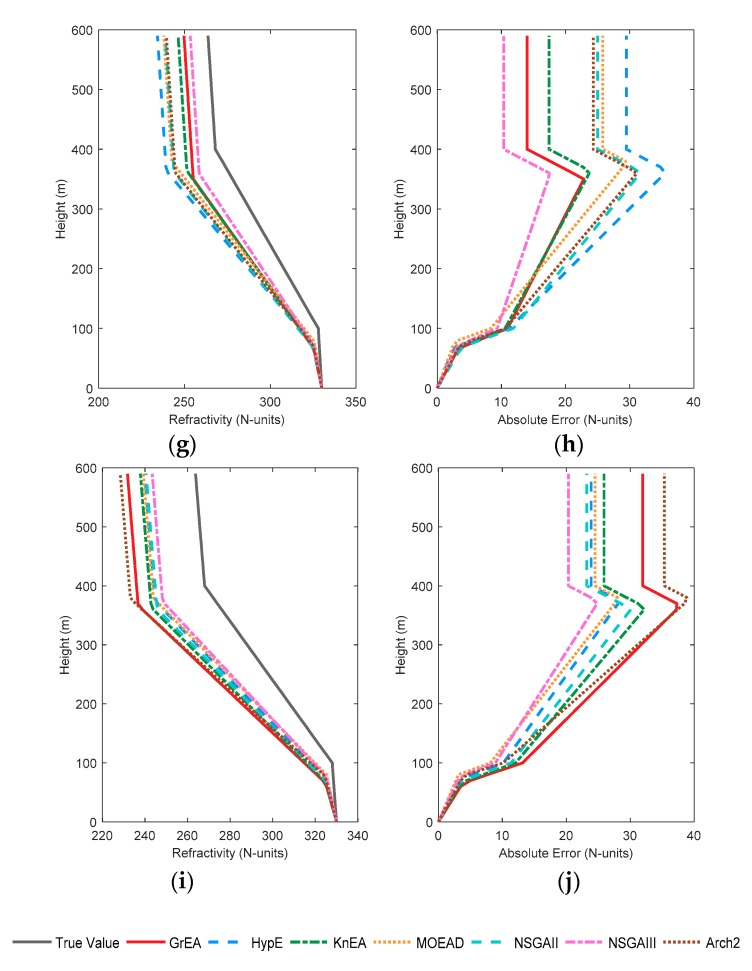
The comparison of simulated and retrieved profiles with 7 test algorithms including transmitting frequency as an inversion parameter. Subfigure (**a**) to (**j**) are the comparison diagram of simulated and retrieved profiles in GPS1, GPS2, GPS3, GPS4, and GPS5, respectively.

Figure 6 shows the spatial distribution of decision space and solution space. When the decision space is five-dimensions, the solution set of MOEAD is still scattered, which is similar to the previous result. However, the decision space of seven algorithms becomes stratified in problem GPS1. Except for MOEAD, the inversion parameters of the algorithms all approximate to the real parameters, and the corresponding HV and IGD values also reflect this phenomenon well. In the solution space, KnEA, marked as green points, is more evenly distributed on the PF. Thus the inversion result of KnEA is improved. Comparing the inversion results of Figure 4 with Figure 7, we can reach similar conclusions. In Figure 6, *f*_2_ of the PF is close to 0.011, which is smaller than the previous approximation value in Section 3.1; other parts of the PF have little change. Therefore, it can be inferred that the retrieved parameters may be improved, as shown by the inversion results in Figure 7.

Figure 7 shows the comparison of simulated and retrieved profiles. When the solution space is two dimensions or four dimensions, the worst inversion results are obtained from HypE, MOEAD and NSGA-II. As the dimensions increase, NSGA-III starts to exhibit slightly better performance. Compared with Figure 4, the inversion result of GrEA improves when the decision space dimension increases in the same dimension of solution space. Similar rules are found for HypE and KnEA. NSGA-III, on the other hand, performs worse in some problems, especially in low-dimensional solution space. The inversion results of Two_Arch2 are not affected by the decision space dimension.

### 3.3. Including Antenna Height and Transmitting Frequency

**Figure 8:**
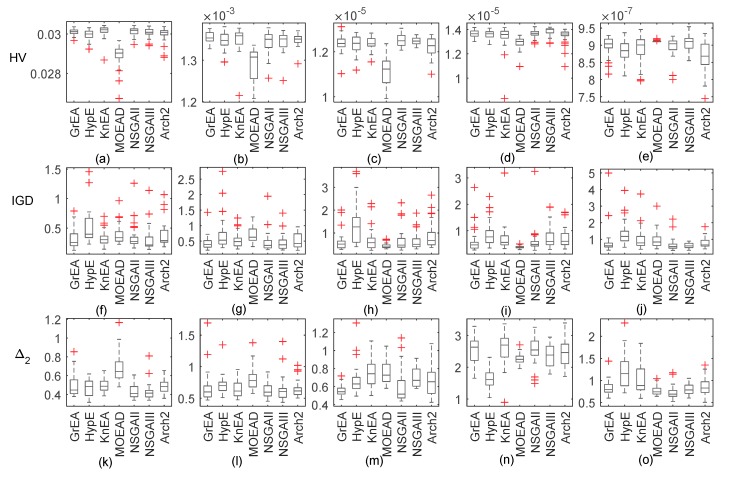
Box plots of the distribution of HV, IGD, and $\Delta_{2}$ values for the inversion problems with 7 test algorithms when considering two factors.Subfigure (**a**) to (**e**) are the boxplots of HV. Subfigure (**f**) to (**j**) are the boxplots of IGD. Subfigure (**k**) to (**o**) are the boxplots of $\Delta_{2}$.

If we obtain a microwave signal with unknown frequency and antenna height, we need to retrieve simultaneously the atmospheric duct parameters, antenna height, and source frequency. Figure 8 shows box plots of all algorithms in six dimensions of decision space. According to the median values, NSGA-II and NSGA-III achieve the best HV and $\Delta_{2}$ values. MOEAD performs poorly in all problems except for GPS5. Compared with the previous values in Section 3.1 and Section 3.2, the abnormal values of the metrics have increased significantly, which further verifies the difficulties with the inverse problem.

**Figure 9:**
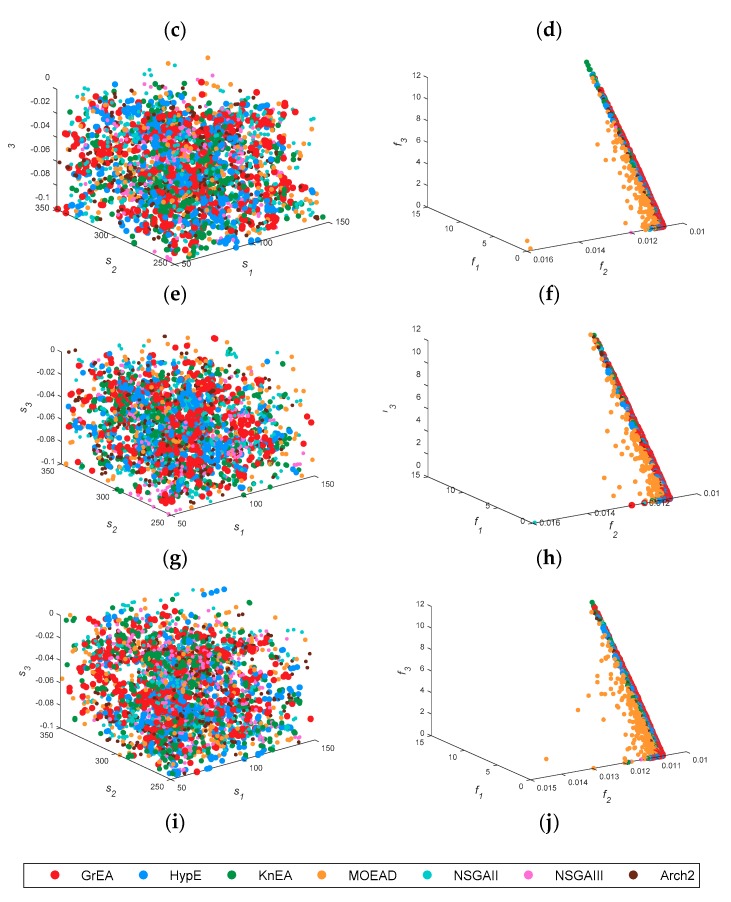
The spatial distribution of decision space and solution space in 7 test algorithms when considering two factors. Subfigure (**a**) to (**j**) are the distribution graph of decision space and solution space in GPS1, GPS2, GPS3, GPS4, and GPS5, respectively.

Figure 9 shows the three-dimensional distribution of decision space and solution space. In general, *S*_1_, *S*_2_ and *S*_3_ are all scattered and untidy in the solution space. In the solution space, *f*_1_, *f*_2_ and *f*_3_ form a rod-shaped structure slanting on the plane *f*_3_ = 0.011. Some yellow points representing MOEAD are scattered on the back of the PF. The solution space becomes convergent and the diversity of decision space is enhanced. This indicates that the disturbance of the inverse problem is enhanced with the increase in decision space dimension. In other words, it is more difficult to obtain the exact value through these multi-objective algorithms.

**Figure 10:**
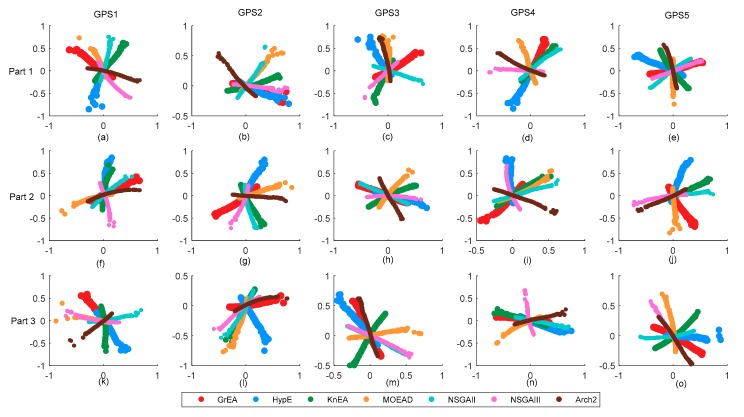
The two-dimensional graph of objective functions. (**a**–**e**) are the results distribution maps of 7 algorithms solving five types of problems in Section 3.1; (**f**–**j**) are the results distribution maps in Section 3.2; (**k**–**n**), and (**o**) are the results distribution maps in Section 3.3.

To illustrate the similarity of objective functions, we use multidimensional scaling method (MSD) to visualize our results. MDS is a powerful statistical method that maps proximity data on pairs of objects into distances between points in a multidimensional space [35]. The space is usually two-dimensional, sometimes also three-dimensional. Here, it is adopted to visualize results’ structure. Figure 10 shows two-dimensional graph of objective functions. We note that Two_Arch2’s results always has different location from other algorithms. In the same scenario, the larger the objective functions dimension is, the smaller the distance among firms are. For example, these results of GPS1 (two-dimensional problem) has larger position distance than these of GPS5 (eight-dimensional problem) in Section 3.1. On ther other hand, these algorithms seems have smaller position distance when increasing known conditions to solve the lower dimensional problems. However, it is obvious that the distances among different algorithms are larger in Section 3.3 than in Section 3.1 for GPS5 problem. The reason of results distribution may be that the increase of constraint conditions does not improve non-uniqueness caused by the number of objective functions.

**Figure 11:**
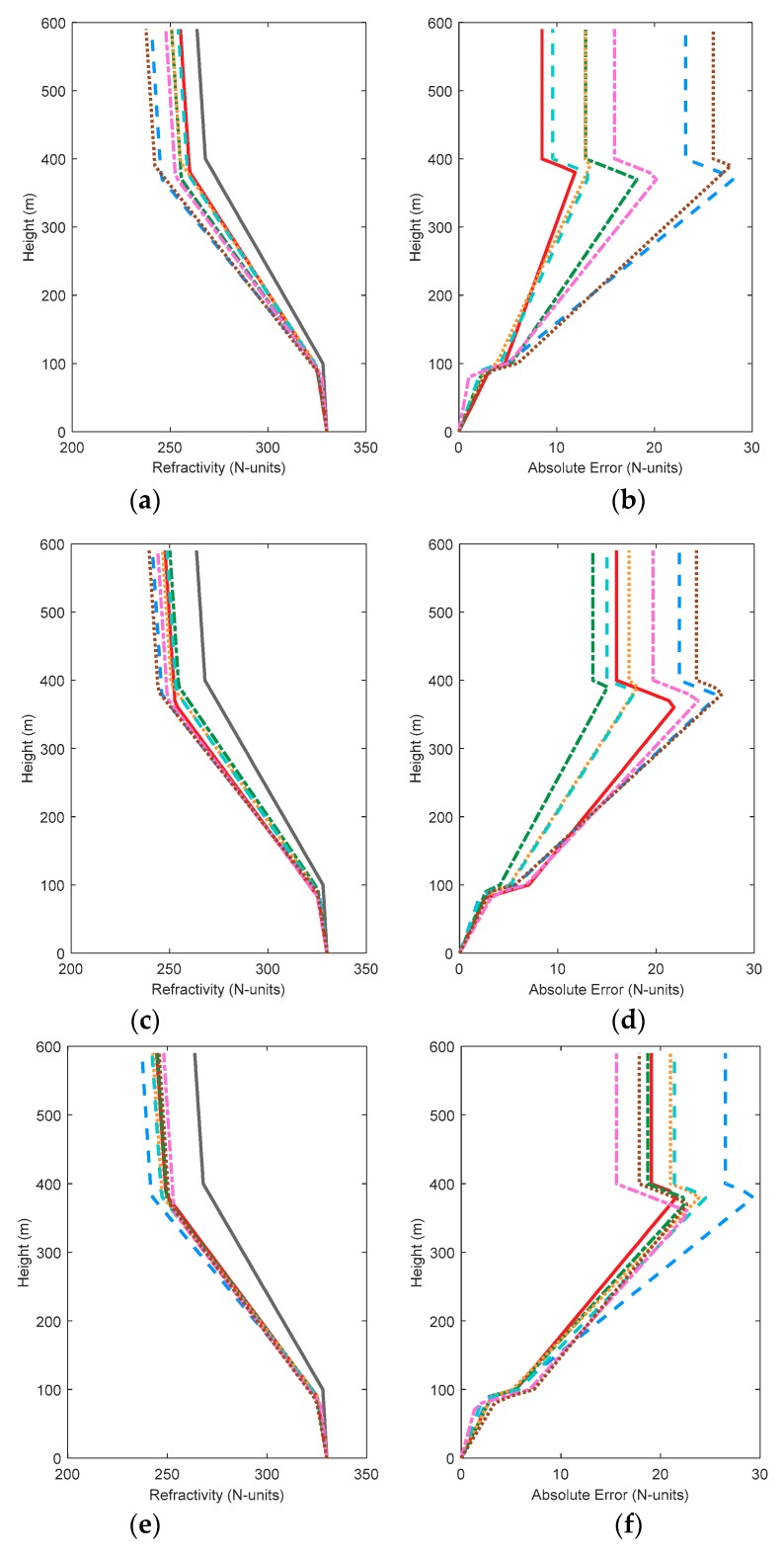

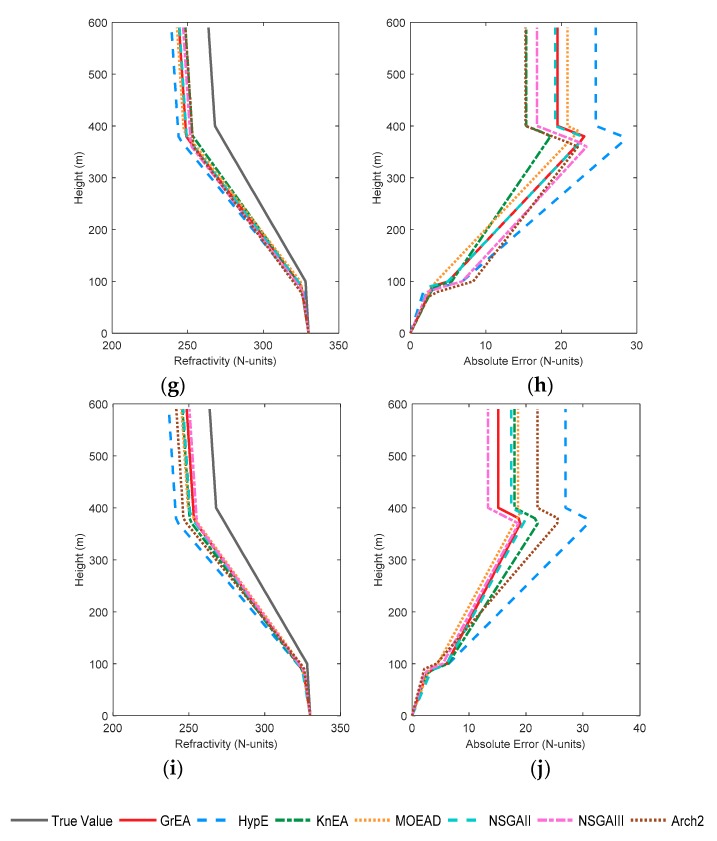
The comparison of simulated and retrieved profiles with 7 test algorithms when considering two factors. Subfigure (**a**) to (**j**) are the comparison diagram of simulated and retrieved profiles in GPS1, GPS2, GPS3, GPS4, and GPS5, respectively.

Figure 11 shows the comparison of simulated and retrieved profiles in eight-dimensional decision space. Two_Arch2 is relatively stable and the inversion results are not improved. However, the absolute error of atmospheric refractivity obtained by the other algorithms is less than the results in Section 3.1 and Section 3.2. This phenomenon is consistent with the solution space distribution. In the solution space distribution, all the cost functions converge on the real PF surface. It can be concluded that although the number of inversion parameters increases, and the difficulties caused by inversion increases, the multi-objective algorithms can still retrieve the real values with their excellent search ability. In problem GPS1, the maximum absolute error obtained by GrEA, MOEAD and NSGA-II hovers at 10 N-units, whereas the inversion results of HypE and Two_Arch2 approach 30 N-units. By comparing NSGA-II and NSGA-III, it can be seen that NSGA-II works well in low-dimensional solution space, especially in two-dimensional space. The maximum absolute error is 12 N-units. NSGA-III always maintains the maximum absolute error around 20 N-units. Comparing the corresponding Figure 11b,d,h and j in the three conditions, the absolute errors of the algorithms, except for GrEA and KnEA, all decrease as the dimension of decision space increases. This indicates that although the dimension of the inversion problem increases, the results obtained can still be improved appropriately. Comparing the results in the three conditions, it can be found that HypE cannot obtain good profiles in all three cases, and may not be applicable when using GPS information to retrieve surface duct.

## 4. Conclusions

The traditional method of detecting atmospheric duct inversion is RFC technology. Based on previous work [15,16], this paper uses the phase delay and propagation loss of ground-based GPS to retrieve the atmospheric refractivity environment with a multi-objective algorithm. Given that different approaches have different search abilities, we divided these tests into three groups according to whether receiving antenna height and transmitting frequency were included as parameters. The performance of seven evolutionary algorithms was evaluated by HV, IGD and $\Delta_{2}$ metrics.

Based on the HV results, NSGA-II remains the top performer in the three test groups, followed by NSGA-III. MOEAD is the worst performer. However, the behavior of the HV and IGD (or $\Delta_{2}$) metrics may differ in some cases, particularly for MOEAD, HypE and GrEA. When no factors are taken into account, KnEA fails to converge retrieved results to real results, and the diversity of the solution space is monotonous. However, when transmitting frequency is considered, KnEA is significantly improved, and the solution space is evenly distributed on the PF. When two factors are considered simultaneously, the solution space converges, and the diversity of the decision space is enhanced. The results show that when the dimension of the decision space increases, the disturbance of the inverse problem is enhanced, and the difficulty of obtaining accurate values through multi-objective algorithms increases.

Comparing the inversion results with the simulation results, it was found that the inversion results are not closer to the real value when compared with other algorithms, even if the algorithm performs well in performance metrics, such as HypE. By contrast, MOEAD and NSGA-III can construct the atmospheric refractivity environment under various conditions, indicating that the two algorithms are more robust. Comparing the three groups of numerical experiments, it was found that when the solution space dimension increases, the multi-objective algorithms can still retrieve the real parameters by virtue of their excellent search ability. However, HypE cannot obtain a good profile in all three cases, suggesting that HypE may not be applicable to such problems.

The multi-objective evolutionary algorithm is used mainly to deal with DTLZ and WFZ problems, and the improved evolutionary algorithm will also be tested on these problems. However, after the computational experiments conducted by Li et al. [25], it was found that these algorithms do not always work well on all test problems. Joint inversion when using ground-based GPS signals is a multi-objective problem. For this problem, future research should aim to improve NSGA-II or NSGA-III to obtain better results. One solution is to search for the knee front instead of the whole PF.

## Figures and Tables

**Table 1:** The parameter settings of the test algorithms. *N* is the population size and *M* is the number of objectives.

Algorithms	Parameter Settings	Categories
GrEA	The grid division: div = 45 for 2 objectives, div = 15 for 4 objectives and 6 objectives, and div = 8 for 8 objectives	C4
HypE	The number of sampling points: 10,000	C2
KnEA	The rate of knee points in population: *K* = 0.6 for 2 objectives and *K* = 0.5 for other conditions.	C3
MOEAD	Neighborhood size T: *N* = 20	C1
NSGA-II	——	C1
NSGA-III	——	C1, C3
Two_Arch2 (abbreviated to Arch2)	The sizes of CA and DA: *N*; the *p* for L*_p_*-norm-based distances: 1/*M*	C5

**Table 2:** Parameter settings for all test problems.

Problems	Inversion Slope *c*_1_ (N-units/m)	Height *h*_1_ (m)	Inversion Slope *c*_2_ (N-units/m)	Height *h*_2_ (m)	Transmitting Frequency (Hz) and Antenna Height (m)
Bounds	[−0.1, 0]	[50, 150]	[−0.4, 0]	[250, 350]	[1200, 1600], [0, 200]
GPS1	−0.02	100	−0.2	300	(1200,20)
GPS2	−0.02	100	−0.2	300	(1200,20), (1300,20)
GPS3	−0.02	100	−0.2	300	(1300,20), (1300,20), (1400,100)
GPS4	−0.02	100	−0.2	300	(1300,20), (1400,20), (1500,20)
GPS5	−0.02	100	−0.2	300	(1200,20), (1300,20), (1400,20), (1500,20)

**Table 3:** HV Results (Mean and SD) of the 7 Algorithms on the GPS Problems. The Top-ranked Algorithms for Each Problem Instance are Highlighted in Grey, the Worst Algorithms Highlighted in Boldface.

Problems	M	D	GrEA	HypE	KnEA	MOEAD	NSGA-II	NSGA-III	Two_Arch2
GPS1	2	4	2.6585 × 10^−2^ (1.2 × 10^−3^)	2.7326 × 10^−2^ (1.4026 × 10^−4^)	2.6453 × 10^−2^ (1.1 × 10^−3^)	**2.6211 × 10^−2^** (5.3499 × 10^−4^)	2.7340 × 10^−2^ (1.6235 × 10^−4^)	2.7221 × 10^−2^ (2.2596 × 10^−4^)	2.7145 × 10^−2^ (2.1758 × 10^−4^)
GPS1	2	5	2.8349 × 10^−2^ (2.3061 × 10^−4^)	2.8137 × 10^−2^ (3.2871 × 10^−4^)	2.8182 × 10^−2^ (4.4032 × 10^−4^)	**2.6666 × 10^−2^** (6.8280 × 10^−4^)	2.8408 × 10^−2^ (2.8335 × 10^−4^)	2.8286 × 10^−2^ (2.1280 × 10^−4^)	2.7963 × 10^−2^ (9.1389 × 10^−4^)
GPS1	2	6	3.0108 × 10^−2^ (1.6557 × 10^−4^)	2.9960 × 10^−2^ (2.5793 × 10^−4^)	3.0153 × 10^−2^ (3.1700 × 10^−4^)	**2.8877 **× **10**^−**2**^ (6.2561 × 10^−4^)	3.0131 × 10^−2^ (2.2698 × 10^−4^)	3.0079 × 10^−2^ (2.3681 × 10^−4^)	2.9978 × 10^−2^ (3.5354 × 10^−4^)
GPS2	4	4	1.0899 × 10^−2^ (1.6894 × 10^−5^)	1.1021 × 10^−2^ (1.6321 × 10^−5^)	1.0525 × 10^−2^ (9.6309 × 10^−5^)	**1.0438 **× **10^−2^** (3.7097 × 10^−5^)	1.1037 × 10^−2^ (1.4758 × 10^−5^)	1.0977 × 10^−2^ (1.6595 × 10^−5^)	1.0838 × 10^−2^ (2.2630 × 10^−5^)
GPS2	4	5	1.1982 × 10^−2^ (2.8865 × 10^−5^)	1.1200 × 10^−2^ (2.4873 × 10^−5^)	1.2076 × 10^−2^ (2.2684 × 10^−5^)	1.1106 × 10^−2^ (4.8637 × 10^−5^)	1.2039 × 10^−2^ (2.1192 × 10^−5^)	1.1923 × 10^−2^ (2.7029 × 10^−5^)	**1.1848 **× **10^−2^** (2.8737 × 10^−5^)
GPS2	4	6	1.3545 × 10^−2^ (1.2246 × 10^−5^)	1.3466 × 10^−2^ (2.0637 × 10^−5^)	1.3482 × 10^−2^ (2.9862 × 10^−5^)	**1.2889 × 10**^−**2**^ (4.0718 × 10^−5^)	1.3434 × 10^−2^ (2.7438 × 10^−5^)	1.3444 × 10^−2^ (2.3390 × 10^−5^)	1.3483 × 10^−2^ (1.3847 × 10^−5^)
GPS3	6	4	9.5495 × 10^−6^ (3.7230 × 10^−7^)	9.4444 × 10^−6^ (4.3702 × 10^−7^)	9.4124 × 10^−6^ (4.4887 × 10^−7^)	9.3182 × 10^−6^ (5.2555 × 10^−7^)	9.5819 × 10^−6^ (3.6928 × 10^−7^)	9.4182 × 10^−6^ (3.2268 × 10^−7^)	**9.1581 × 10^−6^** (6.3368 × 10^−7^)
GPS3	6	5	8.1222 × 10^−6^ (3.5643 × 10^−7^)	8.3087 × 10^−6^ (2.5313 × 10^−7^)	**7.2750 **× **10**^−**6**^ (1.5623 × 10^−6^)	8.0208 × 10^−6^ (9.1172 × 10^−8^)	8.2473 × 10^−6^ (1.7443 × 10^−7^)	8.2147 × 10^−6^ (1.4555 × 10^−7^)	7.9161 × 10^−6^ (4.3874 × 10^−7^)
GPS3	6	6	1.2366 × 10^−5^ (3.7119 × 10^−7^)	1.2321 × 10^−5^ (3.9267 × 10^−7^)	1.2389 × 10^−5^ (2.8948 × 10^−7^)	**1.1170 **× **10^−5^** (6.4066 × 10^−7^)	1.2508 × 10^−5^ (2.7469 × 10^−7^)	1.2483 × 10^−5^ (1.4860 × 10^−7^)	1.2263 × 10^−5^ (4.1432 × 10^−7^)
GPS4	6	4	1.0766 × 10^−5^ (4.0376 × 10^−7^)	1.0877 × 10^−5^ (3.7687 × 10^−7^)	9.6252 × 10^−6^ (2.0121 × 10^−6^)	**8.9592 × 10^−6^** (1.0565 × 10^−6^)	1.0595 × 10^−5^ (4.5682 × 10^−7^)	1.1130 × 10^−5^ (1.4302 × 10^−7^)	1.0066 × 10^−5^ (8.2339 × 10^−7^)
GPS4	6	5	7.9950 × 10^−6^ (4.3028 × 10^−7^)	8.6230 × 10^−6^ (2.7246 × 10^−7^)	**6.3411 **× **10^−5^** (2.0116 × 10^−6^)	8.1312 × 10^−6^ (4.2131 × 10^−7^)	8.4375 × 10^−6^ (2.3223 × 10^−7^)	8.4638 × 10^−6^ (1.3295 × 10^−7^)	8.0950 × 10^−6^ (3.8519 × 10^−7^)
GPS4	6	6	1.3640 × 10^−5^ (2.9747 × 10^−7^)	1.3610 × 10^−5^ (3.2202 × 10^−7^)	1.3367 × 10^−5^ (1.0586 × 10^−6^)	**1.2886**×**10^−5^** (5.5139 × 10^−7^)	1.3668 × 10^−5^ (3.3885 × 10^−7^)	1.3822 × 10^−5^ (4.2498 × 10^−7^)	1.3465 × 10^−5^ (6.4182 × 10^−7^)
GPS5	8	4	4.5080 × 10^−7^ (4.2434 × 10^−8^)	4.9980 × 10^−7^ (1.7298 × 10^−8^)	**3.7706 **× **10**^−**7**^ (1.2623 × 10^−7^)	4.4763 × 10^−7^ (2.0364 × 10^−8^)	4.9074 × 10^−7^ (1.5488 × 10^−8^)	4.3703 × 10^−7^ (6.8565 × 10^−8^)	4.6521 × 10^−7^ (2.9975 × 10^−8^)
GPS5	8	5	6.2653 × 10^−7^ (5.8938 × 10^−8^)	6.636 × 10^−7^ (5.3241 × 10^−8^)	**5.7397 **× **10^−7^** (1.3440 × 10^−7^)	5.9696 × 10^−7^ (2.0599 × 10^−8^)	6.4617 × 10^−7^ (4.7842 × 10^−8^)	6.5795 × 10−7 (2.4359 × 10^−8^)	5.9994 × 10−7 (5.2125 × 10^−8^)
GPS5	8	6	8.9834 × 10^−7^ (2.7039 × 10^−8^)	8.8444 × 10^−7^ (3.1179 × 10^−8^)	8.9006 × 10^−7^ (3.9642 × 10^−8^)	9.1153 × 10^−7^ (2.2031 × 10^−9^)	8.9607 × 10^−7^ (2.8872 × 10^−8^)	9.0699 × 10^−7^ (2.2425 × 10^−8^)	**8.6747 **× **10**^−**7**^ (4.7054 × 10^−8^)

**Table 4:** Results (Mean and SD) of the 7 Algorithms on the GPS Problems. The Top-ranked Algorithms for Each Problem Instance are Highlighted in Grey, the Worst Algorithms Highlighted in Boldface.

Problems	M	D	GrEA	HypE	KnEA	MOEAD	NSGA-II	NSGA-III	Two_Arch2
GPS1	2	4	0.5532 (0.1249)	**0.6959** (0.1768)	0.5667 (0.1090)	0.6142 (0.1200)	0.5644 (0.1489)	0.5406 (0.1580)	0.6288 (0.1513)
GPS1	2	5	0.5247 (0.1210)	**0.6760** (0.1905)	0.5690 (0.1300)	0.6518 (0.1473)	0.5228 (0.1058)	0.5314 (0.1253)	0.5318 (0.0878)
GPS1	2	6	0.5031 (0.1238)	0.4727 (0.0872)	0.4978 (0.0707)	**0.6805** (0.1607)	0.4279 (0.0714)	0.4305 (0.0915)	0.4879 (0.0679)
GPS2	4	4	0.5624 (0.1260)	0.6459 (0.1151)	0.6535 (0.1688)	**0.8319** (0.1605)	0.4927 (0.0657)	0.5685 (0.1145)	0.6069 (0.1652)
GPS2	4	5	0.6610 (0.1435)	0.8141 (0.2557)	0.7091 (0.1587)	**0.8317** (0.1570)	0.6615 (0.1898)	0.6568 (0.1616)	0.6876 (0.1553)
GPS2	4	6	0.6638 (0.2484)	0.7159 (0.1517)	0.6581 (0.1344)	**0.8238** (0.1888)	0.6289 (0.1137)	0.6469 (0.1965)	0.6553 (0.1374)
GPS3	6	4	0.7549 (0.2221)	**1.1262** (0.3770)	0.7968 (0.2498)	0.6521 (0.0750)	0.7781 (0.2516)	0.7835 (0.2078)	0.9112 (0.2722)
GPS3	6	5	0.7158 (0.2150)	**1.0103** (0.3764)	0.7243 (0.1344)	0.5938 (0.0489)	0.7121 (0.2816)	0.6588 (0.0937)	0.7306 (0.1435)
GPS3	6	6	0.5558 (0.0609)	0.6736 (0.1674)	0.7556 (0.1466)	**0.7643** (0.1450)	0.5958 (0.1791)	0.7020 (0.1076)	0.6620 (0.1680)
GPS4	6	4	0.7356 (0.2319)	**0.9056** (0.2345)	0.8331 (0.2543)	0.6157 (0.0396)	0.7662 (0.2418)	0.8304 (0.2317)	0.8278 (0.2028)
GPS4	6	5	0.6563 (0.1441)	0.6673 (0.1811)	**0.8381** (0.2543)	0.6636 (0.0618)	0.7548 (0.1819)	0.7102 (0.1016)	0.7586 (0.2310)
GPS4	6	6	2.5250 (0.3814)	1.6106 (0.3036)	**2.6256** (0.4999)	2.2671 (0.1551)	2.5251 (0.4160)	2.3696 (0.3275)	2.4804 (0.4206)
GPS5	8	4	0.6806 (0.0966)	0.8271 (0.2138)	**1.0168** (0.3198)	0.9126 (0.1148)	0.6615 (0.0663)	0.7255 (0.0993)	0.6372 (0.1070)
GPS5	8	5	0.8639 (0.3150)	**1.1382** (0.2753)	0.9790 (0.2757)	0.9684 (0.2279)	0.7955 (0.1982)	0.7811 (0.0913)	0.8788 (0.1610)
GPS5	8	6	0.8459 (0.1712)	**1.1966** (0.3723)	1.0193 (0.3186)	0.7708 (0.1000)	0.7309 (0.1509)	0.8055 (0.1254)	0.8549 (0.2116)
